# Cumulative Betel Quid Chewing and the Risk of Significant Liver Fibrosis in Subjects With and Without Metabolic Syndrome

**DOI:** 10.3389/fnut.2022.765206

**Published:** 2022-02-11

**Authors:** Yu-Tsung Chou, Zih-Jie Sun, Wei-Chen Shen, Yi-Ching Yang, Feng-Hwa Lu, Chih-Jen Chang, Chung-Yi Li, Jin-Shang Wu

**Affiliations:** ^1^Department of Health Management Center, National Cheng Kung University Hospital, College of Medicine, National Cheng Kung University, Tainan, Taiwan; ^2^Department of Family Medicine, National Cheng Kung University Hospital, College of Medicine, National Cheng Kung University, Tainan, Taiwan; ^3^Department of Family Medicine, National Cheng Kung University Hospital Dou-Liou Branch, College of Medicine, National Cheng Kung University, Yunlin, Taiwan; ^4^Department of Family Medicine, College of Medicine, National Cheng Kung University, Tainan, Taiwan; ^5^Community Healthcare Center, National Cheng Kung University Hospital, College of Medicine, National Cheng Kung University, Tainan, Taiwan; ^6^Department of Geriatric Medicine, School of Medicine, College of Medicine, National Cheng Kung University, Tainan, Taiwan; ^7^Department of Family Medicine, Ditmanson Medical Foundation Chia-Yi Christian Hospital, Chiayi, Taiwan; ^8^Department of Public Health, College of Medicine, National Cheng Kung University, Tainan, Taiwan; ^9^Department of Public Health, College of Public Health, China Medical University, Taichung, Taiwan

**Keywords:** betel quid, *Areca catachu*, liver fibrosis (LF), metabolic syndrome, hepatotoxicity

## Abstract

**Background:**

Betel quid chewing is associated with metabolic disorders, oral cancer, cardiovascular disease, and chronic liver diseases. Metabolic syndrome (MetS) is also a factor associated with liver fibrosis, cirrhosis, and hepatocellular carcinoma (HCC). However, studies on the relationship between betel quid and liver fibrosis while also considering MetS are lacking. The aim of this study was thus to investigate the association of betel quid chewing and liver fibrosis with MetS.

**Methods:**

A total of 9,221 subjects were enrolled after excluding subjects <18 years of age, with past history of chronic liver diseases, cancer, significant alcohol consumption, and incomplete data. Betel nut chewing habit was classified into three groups: none, former-chewing, and current-chewing, and cumulative exposure was calculated by multiplying the duration with the quantity. Liver fibrosis was evaluated based on the NAFLD fibrosis score (NFS), which is a composite score of age, hyperglycemia, BMI, platelet count, albumin, and the AST/ALT ratio. Significant liver fibrosis was defined as NFS ≥-1.455.

**Results:**

After adjusting for other variables, MetS was positively associated with significant liver fibrosis. Subjects with both MetS and betel quid chewing had a higher associated risk of significant liver fibrosis than those with neither MetS nor betel quid chewing (adjusted OR: 3.03, 95% CI: 2.04–4.50, *p* < 0.001). Betel quid chewing was associated with significant liver fibrosis (adjusted OR: 2.00, 95% CI: 1.14–3.49, *p* = 0.015) in subjects with MetS, but not in subjects without.

**Conclusion:**

Metabolic syndrome increased the associated risk of significant liver fibrosis. Cumulative betel quid exposure increased the associated risk of significant liver fibrosis in subjects with MetS, but not in subjects without.

## Introduction

Betel quid chewing has been recognized as a traditional oriental habit in South and Southeast Asia as well as the Asia-Pacific region (1, 2) for hundreds of years. Among different ethnic groups, the betel quid was used in a variety of fields. In the practice of Chinese medicine, the betel quid was used to treat parasitic infection or illness of digestive tracts such as dyspepsia and constipation (3). In ancient Indian culture, the practice of Ayurveda also used betel quid to treat headaches, fever, and rheumatism (3). Till now, betel quid chewing remains common in tropical Asia for different reasons, including social customs and cultural rituals, or is simply used as a psychoactive substance (2, 4). Of note, the increased usage of betel quid was also found among Asian immigrants in western countries recently, and the betel quid is known to be the fourth most commonly used psychoactive substance worldwide (5–7). In Taiwan, the betel quid chewing is commonly practiced by wrapping betel nuts (nuts of *Areca catechu*) with the leaves of *Piper betle*, with additives such as cardamom, catechu, slaked lime, cloves, traditional spices, and other flavorings (8, 9). Despites its popularity, the betel quid chewing is associated with an increased risk of oral cancer (4, 10) as well as several metabolic disorders, including obesity, hypertension, metabolic syndrome (MetS), diabetes, and cardiovascular disease (11, 12). Several previously reported studies have also demonstrated the relationship between and betel quid chewing and liver diseases such as liver fibrosis, liver cirrhosis, and even hepatocellular carcinoma (HCC) (13–15). Considering the harmful effect of betel quid chewing on human health and the negative impact of *A. catechu* on the environment, especially on soil and water conservation, cultivation of *A. catechu* is being discouraged by Taiwan government (16).

Metabolic syndrome is a composite of a cluster of metabolic derangements and is considered a common health problem in the western population (2) as well as in the Asia-Pacific area (17). In spite of the fact that the definition might differ among organizations, the essential components of MetS include hypertension, glucose intolerance, central obesity, and dyslipidemia (18). It is well-known that the presence of MetS is related to an elevated risk of hypertension, type 2 diabetes mellitus (DM), cardiovascular complication, and mortality (19). In addition, MetS is highly associated with non-alcoholic fatty liver diseases such as non-alcoholic fatty liver disease (NAFLD), non-alcoholic steatohepatitis (NASH), liver fibrosis, liver cirrhosis, and even HCC (20–23).

Liver fibrosis occurs in most types of chronic liver diseases. The stage of liver fibrosis is related to liver-related comorbidities, such as liver cirrhosis, hepatic failure, and HCC (24, 25). It was demonstrated that the liver biopsy was the gold standard for diagnosis of liver fibrosis, but the procedure is both costly and has a risk of severe complications (26). Therefore, scoring systems for non-invasive evaluation of liver fibrosis have been designed. For example, the NAFLD fibrosis score (NFS) is well-validated and commonly used for evaluation of liver fibrosis in clinical practice (26, 27).

According to previous studies, both betel quid chewing and MetS have both been found to be associated with the development of liver fibrosis (13, 15, 23). In addition, NAFLD, which was considered as continuum, or precursor of MetS (28, 29), was found to be associated with liver fibrosis in betel quid chewer in one recent study (15). However, the impacts of betel quid chewing and MetS on liver disease have never been considered concomitantly in previous studies (13, 15, 23). Therefore, the aim of this study was to evaluate the relationship between betel quid chewing and significant liver fibrosis with MetS.

## Methods

### Study Population

**Figure 1:**
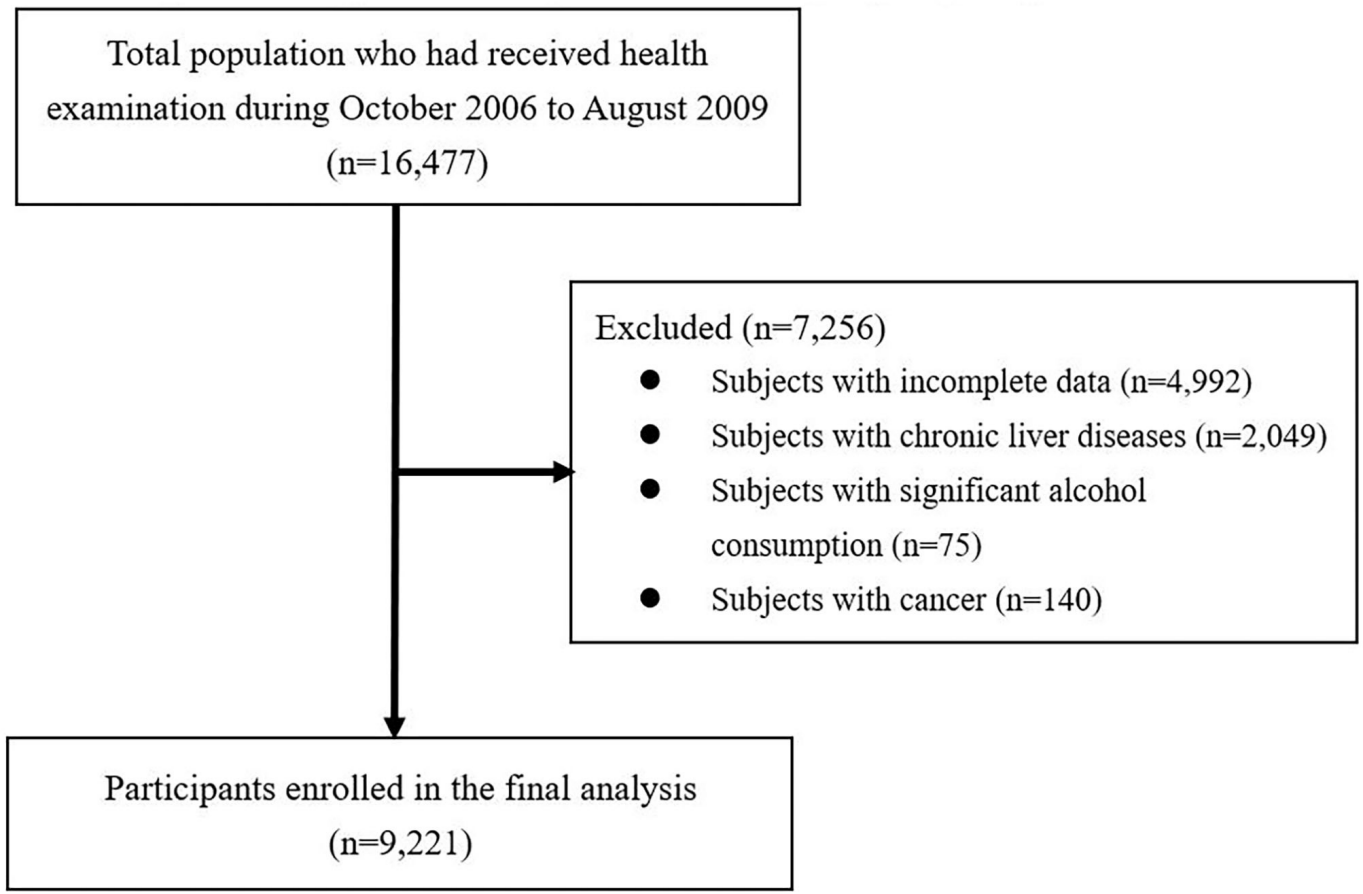
Flow diagram showing the exclusion process for selecting eligible participants.

The participants in this study were recruited from patients who visited the Health Examination Center at National Cheng Kung University Hospital (NCKUH) for self-motivated physical checkups, from October 2006 to August 2009. Initially, a total of 16,477 subjects aged ≥18 years were enrolled in the database. After excluding the subjects with incomplete data (incompletion of the questionnaire or missing variables), a past history of chronic liver diseases (such as hepatitis B, hepatitis C, autoimmune, and drug-related liver disease), cancer, or significant alcohol consumption (subjects with alcohol consumption ≥140 g/week in both genders) (30), a total of 9,221 subjects were included in the final analysis. The exclusion process of participants is shown in Figure 1. An analysis of the decoded secondary data was performed, and the study protocol was approved by NCKUH's Institutional Review Board B-ER-108-326.

### Measures

In order to obtain basic demographic information, all the subjects were asked to complete the standardized self-reported questionnaire that included information on current medication use, past history of chronic diseases, and lifestyle habits, such as betel quid chewing, smoking, alcohol consumption, and regular exercise. Betel quid chewers were categorized into three types such as current chewers, former chewers, and non-chewers. Current chewers were defined as those who had chewed betel quid at least once per week in the previous 6 months (9). Participants who had chewed betel quid but had discontinued using it at least 6 months prior to the health checkup were defined as former chewers. We also collected the information for both duration (years) and quantity (pieces/day) of betel quid use, and then calculated the cumulative exposure of betel quid by multiplying the duration by the quantity (in piece-years). Participants who had smoked at least one pack/month in the previous 6 months were defined as current smokers. Current alcohol consumption was defined as at least one drink per week in the previous 6 months. Regular exercise was defined as those who had exercised vigorously for a minimum of 20 min at least three times per week in the previous 6 months.

Body weight (to the nearest 0.1 kg) and body height (to the nearest 0.1 cm) were measured, and body mass index (BMI) was defined as weight (kg)/height (m^2^). Obesity was defined as BMI ≥27 kg/m^2^ according to the guidelines suggested by the Department of Health in Taiwan (31). With the subject in a standing posture, we measured each participant's waist circumference (WC) between the lower rib margin and the iliac crest at the end of a normal expiration (to the nearest 0.1 cm). After resting for at least 10 min in the supine position, right brachial systolic blood pressure (SBP) and diastolic blood pressure (DBP) were obtained for each participant. Hypertension was defined as medical history of hypertension, SBP ≥140 mmHg, or DBP ≥90 mmHg (32). After overnight fasting for 10 h, each participant underwent a blood draw for the biochemical examinations, including fasting glucose, total cholesterol (TC), triglyceride (TG), high-density lipoprotein cholesterol (HDL-C), glycated hemoglobin (HbA1c), alanine aminotransferase (ALT), aspartate aminotransferase (AST), and platelet count. To test glucose tolerance, each participant was asked to drink 250 ml of a glucose solution containing 75 g of anhydrous glucose, and a blood sample to measure post-load glucose was obtained 2 h after the participant consumed the solution. Diabetes mellitus was defined as a past history of diabetes, FPG level ≥126 mg/dl, a 2-h post-load glucose level ≥200 mg/dl, or HbA1c ≥6.5% (33).

The definition of MetS was established based on the statement from the International Diabetes Federation (34). The participants were diagnosed with MetS if a participant had central obesity (defined as WC ≥80 cm in women or ≥90 cm in men) plus ≥2 of the following conditions: (1) FPG ≥100 mg/dl or previously diagnosed Type 2 DM; (2) BP ≥130/85 mmHg or under treatment of previously diagnosed hypertension; (3) TG ≥150 mg/dl or on medication for hypertriglyceridemia; (4) HDL-C <40 mg/dl in men or <50 mg/dl in women. For non-invasive evaluation of liver fibrosis, NFS was further calculated as NFS = −1.675 + (0.037 ^*^ age) + (0.094 ^*^ BMI) + (1.13 ^*^ hyperglycemia) + (0.99 ^*^ AST/ALT ratio) – (0.013 ^*^ platelet count) – (0.66 ^*^ albumin) and the cutoff value for defining significant liver fibrosis was subjects with NFS ≥-1.455 (35).

### Statistical Analysis

We used SPSS software (version 22.0, SPSS, Inc., Chicago, IL) for data analysis. Continuous variables were expressed as the means ± standard deviations and categorical variables were presented as numbers (percentages). In univariate analysis, independent *t*-test were performed for the comparison of continuous variables and the Pearson's chi-square analysis were performed for categorical variables between two groups. In multivariate analysis, initially we investigated the association of betel quid chewing status and MetS with significant liver fibrosis by logistic regression model. Then subgroup analysis was performed for the relationship between betel quid chewing status and significant liver fibrosis in subjects with and without MetS. The adjustment variables included age, obesity, gender, DM, hypertension, TG HDL-C levels, regular exercise, and current alcohol consumption. The odds ratio (OR) and 95% confidence interval (CI) of MetS with significant liver fibrosis in association with betel quid chewing status were estimated from the regression coefficient and its standard error of logistic regression model. The level of statistical significance was set at an α-level of 0.05.

## Results

The comparisons of the demographic characteristics of the subjects with or without presence of MetS is given in Table 1. Among all the subjects recruited (*n* = 9,221), 2,240 (32.1%) participants met the diagnosis of MetS, and 1,757 (19.1%) participants were diagnosed with significant liver fibrosis (defined as NFS ≥-1.455). The subjects with MetS were older (mean age: 53.7 ± 11.8 vs. 46.8 ± 12.5 years) and more male-predominant (male: 68.7 vs. 56.6%) when compared to those without MetS. In addition, those with MetS had a higher WC, BMI, blood pressure, FPG, 2-h PG, HbA1c, TC, TG, ALT, AST, creatinine, uric acid, and NFS, while their HDL-C levels were significantly lower than that of those without MetS. The prevalence of hypertension (48.2 vs. 12.1%), DM (35.8 vs. 6.5%), significant liver fibrosis (37.0 vs. 13.3%), betel quid chewing (including the duration, quantity, and cumulative betel quid exposure), cigarette smoking, and alcohol consumption were also found to be higher in subjects with MetS.

**Table 1:** Comparisons of participants' clinical characteristics among subjects with or without metabolic syndrome.

**Variables**	**Metabolic syndrome**		***p*-Value**
	**No (*n* = 6,981)**	**Yes (*n* = 2,240)**	
Age, years	46.8 ± 12.5	53.7 ± 11.8	<0.001
<40 years	2,070 (29.7%)	266 (11.9%)	
40–60 years	3,918 (56.1%)	1,314 (58.7%)	
>60 years	993 (14.2%)	660 (29.5%)	
Male	3,948 (56.6%)	1,538 (68.7%)	<0.001
BMI, kg/m^2^	23.3 ± 3.1	27.1 ± 3.2	<0.001
Obesity	735 (10.5%)	1,050 (46.9%)	<0.001
SBP, mmHg	113.7 ± 15.3	129.5 ± 18.3	<0.001
DBP, mmHg	67.3 ± 10.0	76.0 ± 11.1	<0.001
FPG, mg/dl	89.0 ± 15.9	111.7 ± 38.1	<0.001
2h-PG, mg/dl	113.0 ± 40.0	160.2 ± 71.2	<0.001
HbA1c, mg/dl	5.57 ± 0.62	6.36 ± 1.44	<0.001
Total cholesterol, mg/dl	195.8 ± 36.8	207.0 ± 39.1	<0.001
Triglyceride, mg/dl	109.1 ± 62.5	214.0 ± 140.9	<0.001
HDL-C, mg/dl	52.1 ± 13.3	39.0 ± 8.7	<0.001
Hypertension	842 (12.1%)	1,080 (48.2%)	<0.001
Diabetes mellitus	452 (6.5%)	802 (35.8%)	<0.001
ALT, U/L	26.3 ± 17.9	40.7 ± 30.2	<0.001
AST, U/L	23.6 ± 10.8	29.1 ± 17.7	<0.001
NFS	−2.79 ± 1.20	−1.91 ± 1.32	<0.001
NFS ≥-1.455	928 (13.3%)	829 (37.0%)	<0.001
Alcohol use, none	5,603 (80.3%)	1,676 (74.8%)	<0.001
Former	312 (4.5%)	151 (6.7%)	
Current	1,066 (15.3%)	413 (18.4%)	
Cigarette smoking, none	5,370 (76.9%)	1,512 (67.5%)	<0.001
Former	616 (8.8%)	286 (12.8%)	
Current	995 (14.3%)	442 (19.7%)	
Exercise ≥3 times/week	839 (12.0%)	228 (10.2%)	0.018
Betel quid chewing, none	6,657 (95.4%)	2,037 (90.9%)	<0.001
Former	236(3.4%)	125 (5.6%)	
Current	88 (1.3%)	78 (3.5%)	
Duration of betel quid use, none	6,657 (95.4%)	2,037 (90.9%)	<0.001
≤ 10 years	228 (3.3%)	122 (5.4%)	
>10 years	96 (1.4%)	81 (3.6%)	
Quantity of betel quid use, none	6,657 (95.4%)	2,037 (90.9%)	<0.001
≤ 5 pieces/day	72 (1.0%)	39 (1.7%)	
>5 pieces/day	252 (3.6%)	164 (7.3%)	
Cumulative betel quid exposure, none	6,657 (95.4%)	2,037 (90.9%)	<0.001
<150 piece-year	228 (3.3%)	127 (5.7%)	
≥150 piece-year	96 (1.4%)	76 (3.4%)	

*Data expressed as mean ± standard deviation or number (percent). SBP, systolic blood pressure; DBP, diastolic blood pressure; FPG, fasting plasma glucose; 2h-PG, 2-h post-load glucose level; HbA1c, glycated hemoglobin; ALT, alanine aminotransferase; AST, aspartate aminotransferase; HDL-C, high-density lipoprotein-cholesterol; NFS, NAFLD fibrosis score*.

Table 2 demonstrates that, in subjects with MetS, there was an elevated risk of significant liver fibrosis (defined as NFS ≥-1.455) in subjects with (crude OR: 3.63, 95% CI: 2.70–4.88, *p* < 0.001) and without betel quid chewing (crude OR: 3.81, 95% CI: 3.40–4.28, *p* < 0.001) using a binary logistic regression model (Mode 1). Model 2 showed that, after adjusting those potential confounders such as age, gender, alcohol consumption, cigarette smoking, and regular exercise, there was still an increased associated risk of liver fibrosis in subjects with MetS who chewed betel quid (adjusted OR: 5.53, 95% CI: 3.91–7.81, *p* < 0.001) and in those with MetS alone (adjusted OR: 2.70, 95% CI:2.37–3.08, *p* < 0.001). Furthermore, in the presence of MetS, the elevated associated risk of liver fibrosis remained significant whether the participant chewed betel quid (adjusted OR: 3.47, 95% CI: 2.31–5.20, *p* < 0.001) or not (adjusted OR: 1.76, 95% CI: 1.46–2.13, *p* < 0.001), even with additional adjustments for obesity, hypertension, diabetes, and TG and HDL-C levels (Model 3). However, in betel quid chewers without MetS, the associated risk of liver fibrosis was not found to be statistically significant either in the univariate or the multivariate analysis of the logistic regression model. In addition, there was also a positive association of older age, obesity, and DM with liver fibrosis while the TG level and current smoking were negatively related to liver fibrosis (data not shown in Table 2).

**Table 2:** Logistic regression model for risk of significant liver fibrosis (defined as NFS ≥-1.455).

	**Model 1**		**Model 2**		**Model 3**	
**Variables**	**OR (95% CI)**	***p*-Value**	**OR (95% CI)**	***p*-Value**	**OR (95% CI)**	***p*-Value**
Metabolic syndrome (–), Betel quid (–)	Reference	–	Reference	–	Reference	–
Metabolic syndrome (–), Betel quid (+)	0.81 (0.57–1.15)	0.237	1.00 (0.57–1.50)	0.999	0.95 (0.62–1.44)	0.789
Metabolic syndrome (+), Betel quid (–)	**3.81 (3.40–4.28)**	**<0.001**	**2.70 (2.37–3.08)**	**<0.001**	**1.76 (1.46–2.13)**	**<0.001**
Metabolic syndrome (+), Betel quid (+)	**3.63 (2.70–4.88)**	**<0.001**	**5.53 (3.91–7.81)**	**<0.001**	**3.47 (2.31–5.20)**	**<0.001**

*NFS, NAFLD fibrosis score; MetS, metabolic syndrome. Model 2: adjusted for sex, age, alcohol consumption, cigarette smoking, exercise; Model 3: adjusted for sex, age, alcohol consumption, cigarette smoking, exercise, obesity, diabetes, hypertension, triglyceride, high density lipoprotein-cholesterol. Bold values denote statistical significance at the p <0.05 level*.

Table 3 shows the relationship between betel quid chewing and liver fibrosis in subjects with and without MetS based on logistic regression model. Model 1 demonstrated that in subjects with Mets, current betel quid chewing was positively associated with liver fibrosis (adjusted OR: 2.11, 95% CI: 1.19–3.76, *p* = 0.011) after adjusting the other variables. In addition, daily betel quid consumption >5 pieces/day (adjusted OR: 1.80, 95% CI: 1.18–2.75, *p* = 0.006), betel quid chewing duration >10 years (adjusted OR: 1.83, 95% CI: 1.07–3.14, *p* = 0.028), and cumulative betel quid exposure ≥150 piece-year (adjusted OR: 1.94, 95% CI: 1.11–3.41, *p* = 0.021) were also related to higher risk of liver fibrosis (shown in Model 2–4, respectively). On the contrary, in subjects without MetS, the relationship between betel quid chewing and liver fibrosis was found to be insignificant.

**Table 3:** Logistic regression model for significant liver fibrosis (NFS ≥-1.455) among patients with and without metabolic syndrome.

	**MetS (–)**		**MetS (+)**	
**Variables**	**OR** **(95% CI)**	***p*-Value**	**OR** **(95% CI)**	***p*-Value**
**Model 1**				
Betel quid former-chewer vs. non chewer	0.86 (0.52–1.44)	0.573	1.44 (0.90–2.31)	0.127
Betel quid current-chewer vs. non chewer	1.40 (0.60–3.28)	0.433	**2.11 (1.19–3.76)**	**0.011**
**Model 2**				
Betel quid use ≤ 5 pieces/day vs. none	0.98 (0.38–2.57)	0.972	1.18 (0.53–2.66)	0.688
Betel quid use >5 pieces/day vs. none	0.96 (0.59–1.57)	0.879	**1.80 (1.18–2.75)**	**0.006**
**Model 3**				
Betel quid use ≤ 10 years vs. none	1.07 (0.64–1.77)	0.799	1.54 (0.94–2.53)	0.086
Betel quid use >10 years vs. none	0.73 (0.31–1.71)	0.468	**1.83 (1.07–3.14)**	**0.028**
**Model 4**				
Betel quid use <150 piece-year vs. none	1.00 (0.60–1.68)	0.992	1.50 (0.93–2.42)	0.096
Betel quid use ≥150 piece-year vs. none	0.88 (0.39–1.98)	0.753	**1.94 (1.11–3.41)**	**0.021**

*FS, NAFLD fibrosis score; MetS, metabolic syndrome.*

^*^All models adjusted for age (40–60 vs. <40 years and >60 vs. <40 years), gender, obesity, hypertension, diabetes, triglyceride, high density lipoprotein-cholesterol, alcohol consumption, cigarette smoking, and exercise.

*Bold values denote statistical significance at the p <0.05 level*.

## Discussion

This study demonstrated that presence of MetS was related to elevated risk of liver fibrosis, which was in consistent with previous studies that illustrated MetS as risk of chronic liver diseases (20–23). More importantly, the results indicated that betel quid chewing and MetS could lead to higher associated risk of significant liver fibrosis. The associated risk of significant liver fibrosis was found to be up to 3.47 times in MetS subjects who chewed betel quid. The betel quid chewing increased the associated risk of liver fibrosis in subjects with MetS when considering the cumulative exposure depending on duration and dose. In contrast, in the absence of MetS, the association of liver fibrosis with betel quid was found to be insignificant. To the best of our knowledge, we believed that this is the first work investigating the association of cumulative betel quid chewing and MetS with liver fibrosis. Previous studies have demonstrated that several ingredients in betel quid, such as safrole and acrecoline, have a toxic effect on hepatic tissue, causing chronic inflammation and progression of liver cirrhosis and even HCC (13, 36–39). Two studies have shown that betel quid chewing had an additive effect on HBV infection and the development of liver cirrhosis (40, 41). Another study demonstrated that in subjects with NAFLD, betel quid chewing was associated with significant liver fibrosis (15). Although both betel quid habit and MetS have shown to be associated with hepatotoxic effects (13–15, 20–23), these two important risk factors have not been considered concomitantly for liver fibrosis as reported in previous works. The results of the present study showed that the cumulative betel quid chewing increased the associated risk of significant liver fibrosis in subjects with MetS, but not in subjects without MetS. Thus, the results provided a research direction for the relationship between betel quid chewing and significant liver fibrosis in people with different metabolic abnormalities.

The mechanism regarding the association of betel quid chewing and MetS with liver fibrosis remains uncertain. The effects of glucotoxicity and lipotoxicity (11, 42), altered cytochrome P450 2E1 (CYP2E1) activity (37, 43–45) and increased oxidative stress (37, 38, 46), might have played crucial roles in this relationship. Previous studies have shown that consumption of betel quid was associated with central obesity (11) as well as impaired insulin signaling and lipid storage (42). Considering that glucotoxicity and lipotoxicity contribute to hepatic fibrogenesis in subjects with MetS, betel quid chewing might exacerbate these metabolic derangements and resulted into the higher risk of liver fibrosis. An another possible reason for the association of betel quid chewing with liver fibrosis among subjects with MetS might be resulted from two hepatotoxic components of betel quid, safrole and arecoline, and increased activity of CYP2E1 (43–45). Studies have shown that the subjects with diabetes (47), obesity (44), or MetS (48), had an increased activity of CYP2E1, which might be associated with both hepatic toxin metabolism (49, 50) and the development of liver fibrosis (43). It was explained that the metabolism of safrole, a toxic compound in betel quid, was mediated by several types of cytochrome P450 isoenzymes and an increased activity of CYP2E1 was favorable for generating 1′-Hydroxysafrole, a more carcinogenic metabolite (36). In addition, it was understood that arecoline could upregulate the activity of CYP2E1 (37), which could further enhance the hepatotoxicity of betel quid. Moreover, it was observed that MetS was associated with elevated ROS production and increased free radical levels, which might induce liver fibrosis (51, 52) and arecoline could deteriorate this fibrogenesis process by depressing the antioxidants in hepatic tissue (46) and by enhancing the production of ROS (37, 38, 46). On the contrary, in subjects without MetS, there were factors such as lower oxidative stress, ROS, and fewer inflammatory cytokines that might have contributed to less hepatic damage. In addition, without collaboration from elevated CYP2E1 activity, the hepatotoxic effects of safrole and arecoline might be attenuated in those without MetS.

In this study, male gender, older age, obesity, and diabetes were positively associated with liver fibrosis. This result was similar to the results reported in previous studies (53–56). It was demonstrated that aging was commonly associated with higher systemic oxidative stress and elevated ROS production, which might be pivotal in damaging the hepatic tissue. In addition, the repair response to hepatic tissue damage was poor in older people as compared to young people (57). The decreased risk of liver fibrosis in females might have been due to the antifibrogenic effect of estrogen (56). Obesity was usually accompanied with the elevated free fatty acid levels and increased insulin resistance (58). Besides, it was discussed that the visceral fat accumulation was related to several pro-inflammatory mediators that might induce not only hepatic steatosis, but also hepatic damage and subsequent liver fibrosis (55, 59). It was connoted that in patients with diabetes, increased TNF-α and leptin levels might provoke the inflammatory pathways that could contribute to hepatic fibrogenesis (60). Although a negative relationship between TG level and liver fibrosis might be explained by impaired TG production due to hepatocellular dysfunction in subjects with advanced liver fibrosis (61), the exact explanation still requires further studies. In this study, the relationship between liver fibrosis and alcohol consumption was found to be insignificant, which might have been because of the reason that the sample of subjects with risky alcohol drinking habits was relatively small and was excluded in this study (30). We observed a negative relationship between current smokers and liver fibrosis, which might be due to health worker effect among smokers (62). An insignificant association of exercise with liver fibrosis was also found in this study. Although only high-intensity aerobic exercise has been found to improve the status of liver fibrosis, the resistance training and moderate-intensity continuous aerobic training have not been observed (63). However, further investigation is required since the detailed information on exercise habits in the subjects was not available in the present study.

The strength and highlight of our study included not only the large sample size but also concomitant consideration of important covariates for liver fibrosis. However, there were also several limitations to our study. First, it was impossible to establish the causal relationship between betel quid chewing, MetS, and liver fibrosis due to the cross-sectional design. Second, the information on betel quid chewing was obtained from a self-reported questionnaire, and potential recall bias was thus totally excluded. Third, all subjects in this study were recruited from among those who had received health examinations in a tertiary medical center, so selection bias might have been an issue. Fourth, since only 16 female used betel quid, and so it was difficult to perform subgroup analysis by gender to examine whether the toxic effect of betel quid differs among males and females or not. Fifth, although the NFS score had advantages of being relative low cost, easy to obtain, and non-invasive, it was still not the gold standard for the diagnosis of liver fibrosis. Further investigation with histologic evidence might be needed to confirm this finding. Finally, although betel quid has been shown to be hepatotoxic in previous study, direct evidence of hepatotoxicity by betel quid's specific ingredients, such as safrole and arecoline were not available in this study. Further studies, either *in vivo* or *in vitro*, might be necessary to justify this hypothesis.

In conclusion, it was suggested that those with MetS had a higher associated risk of significant liver fibrosis. It was found that cumulative betel quid exposure increased the associated risk of significant liver fibrosis in subjects with MetS, In contrast, it was found that the relationship between betel quid chewing and liver fibrosis was insignificant in subjects without MetS.

## Data Availability Statement

The original contributions presented in the study are included in the article/Supplementary Material, further inquiries can be directed to the corresponding author.

## Ethics Statement

The studies involving human participants were reviewed and approved by Institutional Review Board of National Cheng Kung University Hospital B-ER-108-326. Written informed consent for participation was not required for this study in accordance with the national legislation and the institutional requirements.

## Author Contributions

Y-TC, Z-JS, W-CS, and J-SW contributed to conception and design of the study. Y-CY, F-HL, C-JC, and J-SW organized the database. Y-TC, C-YL, and J-SW performed the statistical analysis. Y-TC wrote the first draft of the manuscript. Z-JS and W-CS wrote sections of the manuscript. All authors contributed to manuscript revision, read, and approved the submitted version.

## Funding

This research was funded by the National Cheng Kung University Hospital, Taiwan (Grant Nos. NCKUH-11003035 and NCKUH-11103034).

## Conflict of Interest

The authors declare that the research was conducted in the absence of any commercial or financial relationships that could be construed as a potential conflict of interest.

## Publisher's Note

All claims expressed in this article are solely those of the authors and do not necessarily represent those of their affiliated organizations, or those of the publisher, the editors and the reviewers. Any product that may be evaluated in this article, or claim that may be made by its manufacturer, is not guaranteed or endorsed by the publisher.
